# Comments on *‘Dietary intervention preserves β cell function in mice through CTCF-mediated transcriptional reprogramming’*

**DOI:** 10.1093/jmcb/mjac043

**Published:** 2022-08-02

**Authors:** Ruo-Ran Wang, Hongxing Fu, Jingya Li, Zhuo-Xian Meng

**Affiliations:** School of Pharmaceutical Science and Technology, Hangzhou Institute for Advanced Study, University of Chinese Academy of Sciences, Hangzhou 310024, China; State Key Laboratory of Drug Research, the National Center for Drug Screening, Shanghai Institute of Materia Medica, Chinese Academy of Sciences, Shanghai 201203, China; Department of Pathology and Pathophysiology and Department of Cardiology of the Second Affiliated Hospital, Zhejiang University School of Medicine, Hangzhou 310058, China; Department of Pharmacy, Shulan (Hangzhou) Hospital Affiliated to Zhejiang Shuren University Shulan International Medical College, Hangzhou 310000, China; School of Pharmaceutical Science and Technology, Hangzhou Institute for Advanced Study, University of Chinese Academy of Sciences, Hangzhou 310024, China; State Key Laboratory of Drug Research, the National Center for Drug Screening, Shanghai Institute of Materia Medica, Chinese Academy of Sciences, Shanghai 201203, China; Department of Pathology and Pathophysiology and Department of Cardiology of the Second Affiliated Hospital, Zhejiang University School of Medicine, Hangzhou 310058, China

Type 2 diabetes (T2D) has become a common chronic disease worldwide. Pancreatic β cell dysfunction, together with insulin resistance, is among the main causes for the pathogenesis of T2D. However, the dynamic changes in the number and function of β cells and the molecular mechanism of irreversible damage in the pathogenesis of T2D are still unclear. A thorough analysis of the mechanism underlying β cell dysfunction will provide a theoretical basis and molecular targets for the development of new and more effective individualized therapies for diabetes.

It is well known that dietary intervention is an effective measure to prevent and treat diabetes. In recent years, many clinical trials have demonstrated that the recovery potential of pancreatic β cell function is a decisive factor in diet intervention for remission and treatment of T2D (Taylor et al., 2018). However, how dietary intervention improves β cell function at different stages of obesity and T2D and its underlying molecular mechanisms remain unclear. Because of the unique anatomy of islets, most studies were based on detecting the changes of insulin levels in blood to reflect the function of β cells. However, the change of insulin level in blood circulation is not only related to the function of islet β cells, but also closely related to the insulin sensitivity of peripheral tissues, and thus it can not reflect the functional change of β cells accurately. Therefore, it is urgent to establish an appropriate and stable animal model to investigate the aforementioned processes and potential mechanisms.

In our recent work (Wang et al., 2022), we established a mouse model exhibiting the compensation-to-decompensation adaptation of β cell function in response to increasing duration of high-fat diet (HFD) feeding. Comprehensive islet functional and transcriptome analyses revealed a dynamic orchestration of transcriptional networks featuring temporal alteration of chromatin remodeling. These sequencing data and conclusions provided an important reference for further study of the dynamic alterations of β cells during the progression of T2D. Further mining and analyzing these data may provide more insights into the dynamic nature and underlying mechanism of islet adaptation during the development of T2D. For example, we noted 45 genes whose expression increased significantly during the compensatory phase but slightly during the decompensatory phase. Interestingly, we found the significant enrichment of pathways related to protein localization to the endoplasmic reticulum (ER), ER-to-Golgi vesical-mediated transport, and intra-Golgi vesicle-mediated transport in these 45 genes. It is possible that these genes are responsible for the irreversible β cell failure at the decompensation stage. Future studies are warranted to test this possibility.

Through systematic functional and multiomics analysis of the dietary intervention model, we found that prediabetic dietary intervention completely rescued β cell dysfunction, accompanied by a remarkable reversal of HFD-induced reprogramming of islet chromatin accessibility and transcriptome. Previous studies have indicated the significant heterogeneity of β cell populations in pancreatic islets (Roscioni et al., 2016; Dominguez-Gutierrez et al., 2019), but the mechanism and the rule of change remain unclear. Our study found that dietary intervention altered the proportion of β cell subpopulations β1–β4 with distinct characteristics of gene expression profiles. However, the mechanism of the functional and proportional changes of the β cell subpopulations in pancreatic islets remains to be explored.

Mechanistically, we identified CTCF as the top candidate driving dietary intervention-induced preservation of β cell function. In addition to its important role as an insulator in the maintenance of high-level chromatin structures, several studies in recent years have suggested that CTCF can also act as a transcription factor, playing a role in regulating gene expression (Braccioli and Wit, 2019). In our study, cleavage under targets and tagmentation (CUT&Tag) analysis showed that dietary intervention ameliorated the functional impairment of T2D islet β cells through CTCF-mediated transcriptional reprogramming. CTCF has been widely reported in previous studies as a regulator of the three-dimensional structure of chromatin, regulating the transcriptional level of downstream genes by regulating enhancer–promoter interactions (Ren et al., 2017). In our study, many of the CTCF-binding regions with altered chromatin accessibility overlapped with known distal enhancer and promoter regions. It is suggested that some of the transcriptomic changes regulated by CTCF in β cells may be related to changes in the three-dimensional structure of chromatin. Therefore, it may be interesting to explore whether the three-dimensional structure of chromatin indeed changes significantly during dietary interventions and whether such changes have any potential implications.

In conclusion, our recent studies systematically delineate a dynamic map of β cell function and gene expression profiles during the onset and development of obesity and T2D, revealing that dietary intervention improves β cell function in T2D. On this basis, we found that the DNA-binding protein CTCF may regulate gene transcription related to β cell function by responding to lipotoxicity and inflammatory signal factors, mediating the protective effect of dietary intervention on β cell function. In addition, our work also raises several questions needed to be resolved (Figure 1).

**Figure 1:**
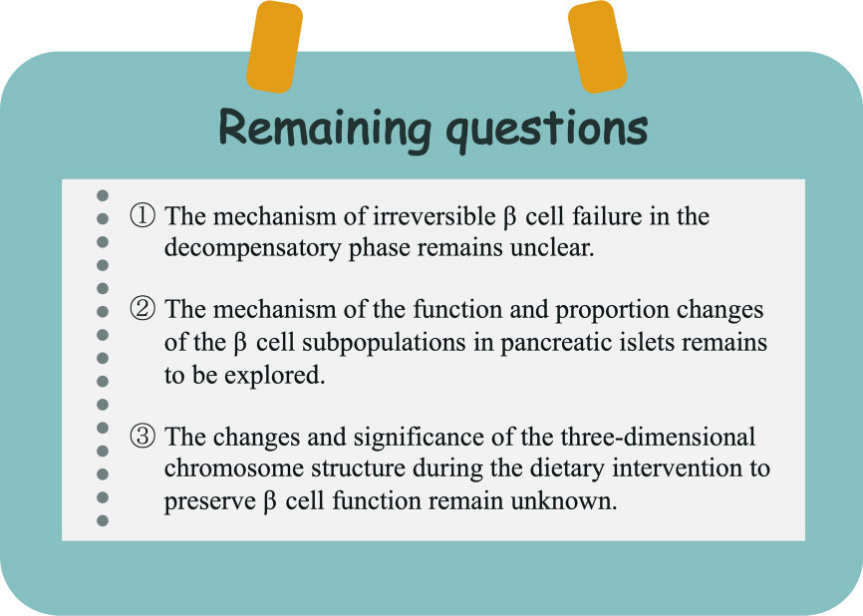
Remaining questions for future studies.

## Notes

*[This work was supported in part by the National Natural Science Foundation of China (91857110, 81722012, 81670740, 82070805, 81870535, and 31870855), the National Key Research and Development Programme of China (2018YFA0800403, 2021YFC20701903, and 2020YFA0803704), Zhejiang Provincial Natural Science Foundation of China (LZ21H070001), the Construction Fund of Key Medical Disciplines of Hangzhou (OO20200055), the Innovative Institute of Basic Medical Sciences of Zhejiang University, and the Fundamental Research Funds for the Central Universities.]*
